# The Spanish flu in Uppsala, clinical and epidemiological impact of the influenza pandemic 1918–1919 on a Swedish county

**DOI:** 10.3402/iee.v4.21528

**Published:** 2014-01-17

**Authors:** Jonas Holtenius, Anna Gillman

**Affiliations:** Section of Infectious Diseases, Department of Medical Sciences, Uppsala University, Uppsala, Sweden

**Keywords:** Spanish influenza, age-specific mortality, autopsy, treatments, economy, 1918 pandemic, Sweden

## Abstract

**Introduction and aim:**

The Spanish flu reached Sweden in June 1918, and at least one-third of the population (then 5.8 million) became infected. Some 34,500 persons (5.9 per 1,000 people) died from influenza during the first year of the pandemic (when acute pneumonia is included, the number of deaths rose to 7.1 per 1,000 people). In this historical look back at the pandemic, our aim was to review the epidemiological impact on the Swedish county of Uppsala, the clinical outcomes and the economic impact on the regional hospital; a relevant backgound to consider the impact of a future virulent pandemic. We also focused on how the pandemic was perceived by the medical community and by health care authorities.

**Methods:**

Health care reports, statistics, daily newspapers, medical journals, and records of patients treated for influenza at the Uppsala Academic Hospital from July 1918 to June 1919 were included in our review.

**Results:**

An influenza related mortality rate of 693 persons (5.1 per 1,000 people) was reported in the Uppsala region from 1918–1919; from July 1918 to June 1919, 384 patients were treated for influenza at the Uppsala Academic Hospital. The first wave peaked in November 1918 with case fatality rates up to 30%; a second wave peaked in April 1919 with a lower rate of mortality. Of the patients treated, a total of 66 died. Of these, 60% were 20–29 years of age, and 85% were less than 40 years old. Autopsy reports revealed pneumonia in 89% of the cases; among these, 47% were hemorrhagic, 18% were bilateral, and 45% had additional extrapulmonary organ involvement. Signs of severe viral disease were documented, but secondary bacterial disease was the primary cause of death in the majority of cases.

**Conclusion:**

The epidemiologic and pathologic results were in accordance with other publications of this time period. The costs of running the hospital doubled from 1917 to 1920 and then reversed by 45%. Today, an influenza pandemic of the same virulence would paralyze health care systems and result in extremely high financial costs and rates of mortality.

In March 1918, there was an outbreak of influenza A in Kansas at the U.S. Army's Camp Funston [now Fort Riley] that later was considered to be the starting point of the great influenza pandemic (1–3). However, there also are reports from China during the winter of 1917–1918 (4) of a disease that could be the same strain of influenza and epidemiologic indications of a mild wave of the influenza pandemic in New York City during February–April 1918 (5). The first cases in Europe probably appeared in the beginning of April 1918 among U.S. soldiers in a military camp close to Bordeaux in southwest France (1).

Because of wartime censorship not much was written about the influenza until May 1918 when the disease reached Spain, a country unaffected by censorship due to its neutrality during the war. The Madrid newspaper *El Sol* first covered the new disease on May 22. When King Alfonso XIII and his cabinet became ill from influenza a week later, world headlines labeled the pandemic ‘the Spanish flu’ (6, 7). However, it was not until the fall of 1918, when the virus had become much more virulent, that the actual pandemic spread globally (8, 9). The Spanish flu was to become one of the worst pandemics in human history. Estimations of the total number of deaths have ranged from 21 million (10) to as high as 100 million, especially when the lack of data from many parts of the world is considered (2, 11). The case fatality rate varied greatly but averaged from 2.5 to 5% globally. However, in some remote locations, the devastation has been estimated as high as 90% (9, 12). In Europe, where the pandemic may have begun and where war was being waged, the overall excess mortality rate varied among countries, averaging 1.1% (13). In southern Europe, influenza related mortality rates have been calculated at approximately 6 per 1,000 people and to 11–12 per 1,000 people including all deaths related to respiratory organs (7, 13, 14).

The Spanish flu affected young adults in particular. For the 1918 pandemic, age related mortality was typically W-shaped (as compared to the typical U-shape of seasonal influenza mortality). With the Spanish flu, there were peaks in infants and the elderly but there also was a third rate peak in the 20–45 age groups (9, 15). Compared with the seasonal influenza rate, the older age groups were spared. It has been estimated that during 1918 persons under 65 years of age comprised 84–99% of excess influenza related mortality totals (16, 17). In major cities in southern Europe, such as Paris and Madrid, the proportion of deaths in the 15–44 age groups that were due to influenza was 66–68% (7).

The first Swedish cases of the pandemic influenza arrived from Norway and Germany late in June 1918. Initially, the spread was slow and was confined to southern Sweden (18). It was not until July 6, 1918, when the steamboat *Torsten* (19) reached Gothenburg carrying passengers from London that the pandemic speeded up and quickly started to spread throughout the country (18). The proportion of the Swedish population infected is estimated at 20–60%, with higher proportions in younger age groups (18, 20–23). For the total Swedish population of 5.8 million people, the number of pandemic influenza deaths between July 1918 and June 1919 is estimated at 34,374 (5.9 per 1,000 people) and at 37,573 (6.5 per 1,000 population) during all three pandemic years – from 1918 to 1920 (18, 24–26). Deaths due to acute pneumonia increased by 35% during the pandemic year 1918–1919. During this same period, the mortality rate for influenza plus acute pneumonia was 7.1 per 1,000 (18, 27); adding deaths due to pulmonary tuberculosis increased the mortality rate to 8.7 per 1,000 people (27). The highest mortality rate was among people of working age. The overall mortality rate among Swedes in the 20–40 age group rose by almost 200%, and the total population life expectancy decreased from 59 years in 1917 to 50 years in 1918 (28). An analysis of the pandemic's impact on the Swedish economy found an increase in short- and medium-term poverty. In addition, with each deceased individual, four poorhouse residents were added because of dependents being left behind (29).

No previous studies have been published that specifically detail the effects of the Spanish flu on the Uppsala region in general, on the health care facilities there, or on the outcome of the clinical cases treated at the regional referral hospital. In order to consider our present health care structures’ capability of supporting a future severe influenza pandemic, we find it interesting to look back at the most severe influenza pandemic from a local historical perspective. In this study, we investigated the clinical, epidemiological, and financial effects of the Spanish flu on the Swedish county of Uppsala and on the regional Uppsala Academic Hospital. Medical and political discussions at the time and the extent to which these discussions influenced health care decision makers were also reviewed.

## Sources and methods

Epidemiologic and demographic information on the health situation in the Uppsala region was collected from historical and modern references and from historical statistics – primarily the yearly reports of the Uppsala County medical officer and the statistic report series published by Statistics Sweden, that country's official statistics.

Death and housing statistics have been collected in Sweden since the 18th century; beginning in 1860, a population census was undertaken every 10 years. The latest population census prior to the pandemic had taken place in 1910 (30). Statistics Sweden obtained mortality figures from church death books where priests registered everyone who died in each parish. After 1911, Statistics Sweden introduced a more detailed reporting system where priests were required to note a cause for deaths undetermined by a physician. Each month a report was sent to the county medical officer who noted whether a cause of death was proven, probable, or (rarely) unknown (18). Following instructions from the Swedish Medical Board (the highest governmental authority supervising the health care system), doctors that ascribed acute pneumonia as cause of death were to determine if the pneumonia was associated with influenza. The cause of death would then be classified as influenza related. However, not all doctors complied and some cases were attributed simply to acute pneumonia (18, 31).

Each year, death statistics were published in a cause of death report series. Beginning in 1914, the *Statistical Yearbook of Sweden* was published containing historical population information and detailed statistics on many matters that were updated annually.

The clinical picture, epidemiologic information, and mortality rates at the Uppsala Academic Hospital were evaluated by studying the charts and autopsy protocols of the hospital's medical ward during the period of July 1, 1918, to June 30, 1919. Patients with a clinical diagnosis of influenza in the chart ledger were selected; their charts were reviewed and records were compiled regarding age, gender, length of time in the hospital, clinical symptoms, and time from initial symptoms to death. Autopsy protocols for deaths were reviewed for pathological findings and for the classified cause of death.

A survey of medical and political discussions was undertaken by reviewing articles regarding the Spanish flu during the time period of July 1, 1918, to June 30, 1919, from the two main Swedish medical journals of the time: *Svenska Allmänna Läkartidningen* and *Hygiea*, the journal of the Swedish Medical Society. The records of the Environmental Health and Safety Committee (*hälsovårdsnämnden*) were reviewed with a focus on public health measures related to the pandemic. To evaluate the economic impact on the hospital, the yearly financial reports of the Uppsala Academic Hospital from 1917 to 1923 were reviewed.

## Results

### Uppsala epidemiology

**Fig. 1:**
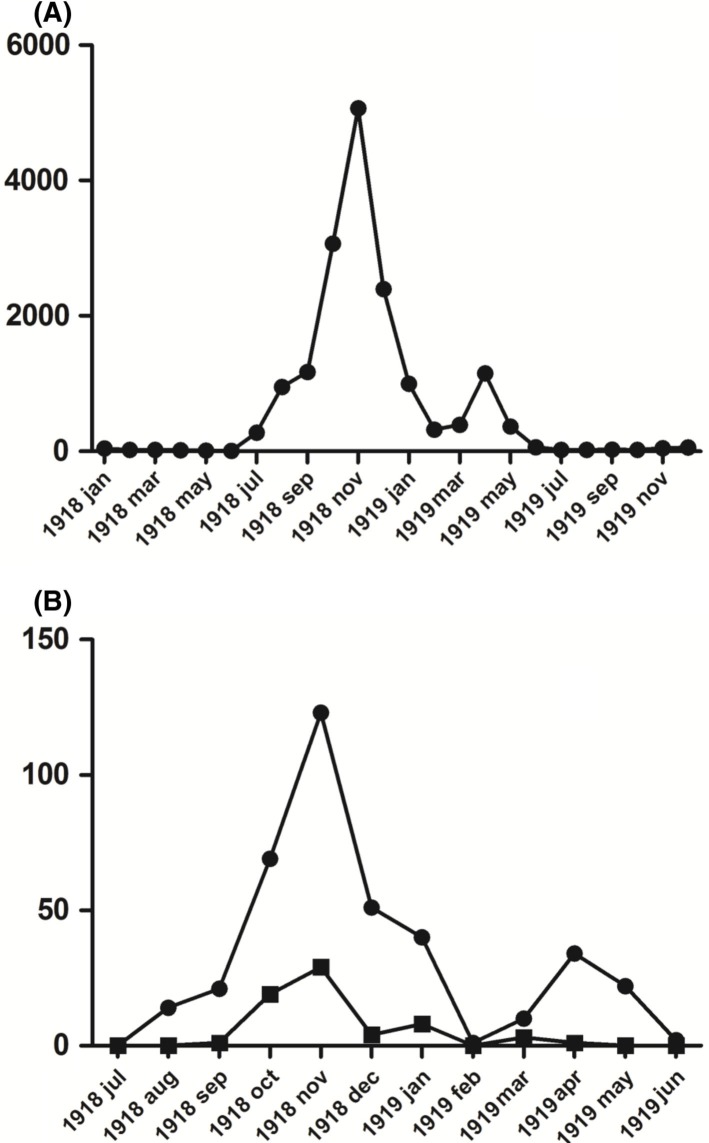
A) The Spanish flu in Uppsala County January 1918 to December 1919 according to the records of the Uppsala County Officer. B) Admitted cases with influenza diagnosis (•) and death cases (■) at the Uppsala Academic Hospital July 1918 to June 1919. Y-axis: Number of reported influenza cases per month.

In 1918, the county of Uppsala had a registered population of 134,622; the majority of residents lived in rural areas and in small villages. The populations of the two cities, Uppsala and Enköping, were 28,877 and 5,938, respectively (32). The pandemic is believed to have been brought to the region by a school teacher from Älvkarleby (in the northern part of the county) who, in July 1918, had visited her family in Gothenburg (33), where the influenza had spread from passengers who had been aboard the *Torsten* (19). Beginning in July, the number of cases rapidly increased and reached a peak in November 1918 when 5,067 cases were recorded in the county, 31% of all recorded influenza cases from July 1918 to June 1919 (33; see Fig. 1). The mortality rate also peaked during November when 69 of the 143 deaths (48%) registered in Uppsala and Enköping occurred (33). A second wave, with a lower mortality rate, peaked in April 1919 with 1,150 recorded cases in the county (33) (Fig. 1). According to Statistics Sweden, there were 693 deaths due to influenza in Uppsala County in 1918–1919 (5.1 per 1,000 people) (18, 24, 25). This mortality rate fell in the lower range among Sweden's 25 counties (4.6–9.7 per 1,000 people, 1918–1919) (18, 29).

### Clinical findings at Uppsala Academic Hospital

In Uppsala Academic Hospital's medical ward, during the period from July 1, 1918, to June 30, 1919, 384 patients were diagnosed with influenza based on the clinical picture. Among them, 49% were women and 51% were men; the average age was 27.

The initial admission with Spanish flu as the primary diagnosis was on August 3, 1918. The first 32 patients admitted survived the disease, and the first influenza related death at the clinic did not occur until October 2. After this, the mortality rate quickly rose to its peak period (from October 30 to November 14) when the case fatality rate for the 88 admitted patients was 32% (Fig. 1B).

During November 1918, when the pandemic was at its peak in Uppsala, 118 of 151 patients (78%) admitted to the medical ward had influenza as their primary clinical diagnosis. During the second peak, in the spring of 1919, the mortality rate was substantially lower – 6% of the 66 cases admitted from March to May (Fig. 1B).

The influenza patients’ most common symptoms were an elevated temperature and a cough but muscle ache, vomiting, fatigue, and headache were other symptoms often described in the clinical records.

**Fig. 2:**
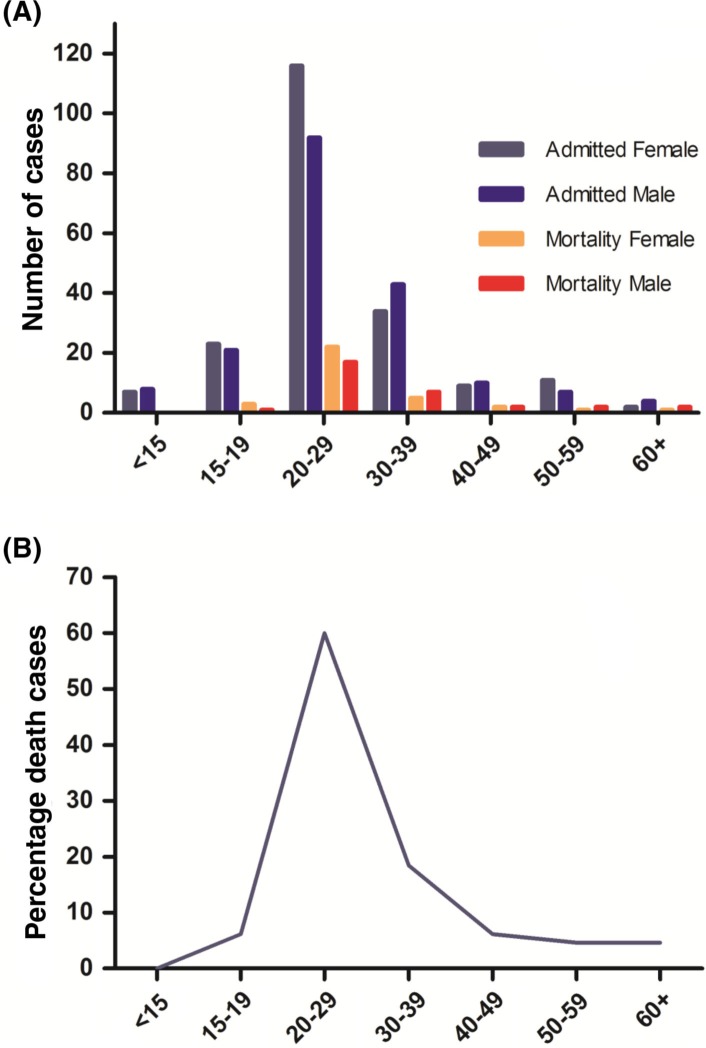
Patients treated for influenza at the Medical Ward at Uppsala Academic Hospital July 1918 to June 1919. A) Age distribution of admitted cases and death cases by gender. B) Age distribution of death cases by percentage, n=66. X-axis: Age groups by years of age

In total, 66 of the 384 patients (17%) who were treated for influenza died at the hospital during the period. Among the deaths, 32 (48%) were female and 34 (52%) male. The age of admitted and fatal cases followed the previously noted pattern with the highest incidence and mortality in the 20–29 age group; 85% of the deaths were of patients younger than 40 (Fig. 2). The duration of symptoms from reported onset until death was 9.6 (SD 3.3) days (range 1–16). The time period from admittance to death was 3.7 (SD 2.2) days (range 1–10).

An autopsy was performed on 57 (86%) of the patients that died. The terminology in the autopsy records was not completely consistent and the primary cause of death not explicitly specified in all cases; 55/57 records were evaluable. The most commonly reported pathological finding was pneumonia, in 49 (89%) of the cases. Of the six where pneumonia was not documented, two cases had endocarditis and four cases had pleural disease. Among the cases with pneumonia findings seven (15%) included empyema, 23 (47%) had hemorrhagic pulmonary disease, nine (18%) had bilateral involvement, and 22 (45%) had involvement of other organs (Table 1). Of those patients with other affected organs (aside from airway disease), nine cases were cardiac, three had spleen inflammation, four had meningitis, two had renal disease, and two had gastric ulceration.

**Table 1:** Autopsy Results for Influenza Cases, Uppsala Academic Hospital, July 1918–June 1919

Autopsy results (n=55)				

Bronchopneumonia 49				Non pulmonary 6 *
Bilateral	Hemorrhagic	Empyema	Extra thoracic	
9	23	7	22	

* Endocarditis (n=2) and pleural disease (n=4). 66 patients died while treated under influenza diagnosis. 57 were autopsied, 55 autopsy records were evaluable.

### Economic impact on Uppsala Academic Hospital

From 1917 to 1920, the total cost of running the Uppsala Academic Hospital doubled (SEK 510,000–1,082,000) and then successively dropped 45% until 1922 (to SEK 827,000). The primary cost increases were in salaries, medical supplies, work materials, and food. After the pandemic period, work materials and food expenses decreased again.

### Review of medical literature

#### Clinical description of the Spanish flu in Sweden

Uncomplicated cases of the Spanish flu were described as similar to seasonal influenza with a quick onset of high fever and malaise. Dry cough was common but, in contrast to the seasonal influenza, nose bleeds often appeared (34). In more serious cases, a grave cyanosis was often present, and this was described as the single most important predictor of a fatal outcome (35).

Many of the patients developed what was described as post influenza pneumonia a few days after the onset of the influenza symptoms. This occurred without any new alarming symptoms and a prolonged duration of elevated temperature was commonly the first indication of a secondary infection (35).

#### Treatment of influenza

In Swedish hospitals, the typical treatment for severe influenza was a combination of cognac, tea, enema, camphor injections, and wrapping in bed linen soaked in cold water. The goal was to induce perspiration, which was believed to reduce mortality (36).

There was no consensus on the effectiveness of the perspiration method, and other therapies were also attempted. These included injections of a serum against diphtheria (37) and syphilis treatments such as neosalvarsan and arsenic (38). Heroin was used as a cough dampening treatment (39). The patent for Aspirin^®^ had expired in 1917, and acetylsalicylic acid was widely used – also in combination with digitalis. Later assessments have suggested that there was an increased mortality among patients treated according to this regimen (40).

#### Causative agent of the Spanish flu

The Spanish flu's causative agent and method of transmission were unknown, and discussions and debates in the medical journals were extensive and lively. There were arguments for transmission by direct contact or by droplet transmission, but fog and vapor also were proposed as contributing to transmission because misty weather seemed to covary with spread of the disease (41). A common theory espoused that the causative agent was a microbe too small for detection under a microscope. Many also believed that the cause was a bacterial agent, primarily the ‘Pfiffer bacillus’ (eventually named *Haemophilus influenzae*) or another bacteria with an ability to transform into different shapes depending on which organ was involved (41, 42). The unusual epidemiological pattern – high mortality in young healthy adults – was a major topic of discussion. Although many believed this pattern was due to a lack of immunity in young adults, compared to those who had encountered the influenza pandemic in 1889 (‘the Russian influenza’) (42), some argued that the causative agent must have evolved in the war's dead and wounded soldiers and therefore developing an avidity for that age group (41).

### Actions taken to stop the pandemic

At the time, influenza was not classified as one of diseases regulated by Sweden's epidemic laws. Therefore, patients in Uppsala who were suffering from the Spanish flu were not treated at the epidemic hospital outside the city center, where other transmissible diseases were handled. This classification and the low mortality during the first months of the outbreak made the health care authorities rather passive in the early stage of the pandemic. The Swedish Medical Board described the pandemic as ‘widespread but of a mild nature’ (43) and did not recommend actions to be undertaken against the spread of the disease during the late summer and early autumn of 1918. However, the passive approach by health authorities was criticized by the Swedish Medical Society (the medical doctors’ association). They argued for active legislation to prevent the spread of the disease, such as prolonged summer vacations for school children, forced home stay for infected workers, and an exercise free week for military recruits recovering from the influenza (44). There were also proposals of fines for sneezing, laughing, and coughing in public areas as well as the branding of infected individuals (45). With the exception of closing dance halls beginning on November 6, no other suggested actions were undertaken in Uppsala because they were considered inefficient by local authorities (46).

## Discussion

We describe how the influenza pandemic (‘the Spanish flu’) reached and affected the Swedish county of Uppsala in 1918–1919 and the outcome of patients treated at the Uppsala Academic Hospital during the first year of the pandemic. The epidemiological impact of the pandemic on Uppsala and on the Uppsala Academic Hospital with two waves of the disease (a more fatal wave that peaked in November 1918 and a milder one that peaked in April 1919) and an age profile with highest incidence of the disease and resulting mortality among young adults, follow the patterns described in the literature during the pandemic's epoch and later (1, 3, 7, 14, 42).

General epidemiologic figures on the effects of the 1918–1919 influenza pandemic are undisputedly approximate; incidence, case fatality rates, and overall excess mortality varied greatly globally between and within countries; much of the information relies on estimates (2, 9, 11–13). The Swedish population and death totals from Statistics Sweden are, however, rather unique, and the figures for numbers of deaths during the pandemic can be reasonably relied upon. There is certainly a proportion of cases that were misclassified as acute pneumonia (not influenza associated) because this diagnosis increased by 35% in the registers during the period, an issue addressed already at the time (44). The number of ‘unknown’ causes of death did not, however, increase during this period (32), which is why the overall statistics for influenza and acute pneumonia appear reliable. The Swedish excess mortality rate of 5.9–6.5 per 1,000 (18, 24–26) is in parity with European figures (7). However, the additional number of deaths due to acute pneumonia (1.2 per 1,000) and pulmonary tuberculosis (1.7 per 1,000) in 1918–1919 appears substantially lower compared to the figures at southern European sites when other respiratory diseases are added to the overall mortality rate (7, 14).

In Uppsala County, the influenza related mortality rate of 5.1 per 1,000 people in 1918–1919 was among the lower figures for Sweden (18, 29). Only 12% of the Uppsala population was documented with influenza, which generates a calculated case fatality rate of 4.2% (24, 25, 33). The true incidence was certainly higher (and, thus, the case fatality rate lower) because many cases, especially uncomplicated ones, must have been undocumented by the county medical officer, and all surveys from the time period indicate up to a 50% incidence rate (20–23). It is probable, however, that Uppsala had slightly fewer and milder influenza cases then other parts of country. The variance in mortality across Sweden was inversely related to the proportion of the population over 40 years of age. Uppsala's somewhat higher average age (32.5% compared to 29.2% of the population over 40 in the most severely affected Swedish county) (18) appears to be the most reasonable explanation for the lower mortality figures. This is in accordance with the age pattern of pandemic influenza cases (5, 15, 17, 47) where a higher average age of a population is associated with lower influenza pandemic related mortality, primarily due to immunity from previous influenza epidemics (18, 47). Uppsala was a relatively wealthy area with an annual per capita income (SEK 609) approximately 20% higher than other comparable Swedish cities (20,000–35,000 inhabitants with an average income of SEK 509) (32) and had correspondingly higher standards of living, which might have influenced the lower mortality rate. Social class per se, however has not been shown to influence mortality but rather location factors related to class (29).

During the period from July 1918 to June 1919, the majority of the 66 patients that died from the Spanish flu at the Uppsala Academic Hospital were in the highly affected age group of young adults; 60% of the deaths were patients in the 20–29 year age group and 85% of all cases occurred in patients under 40 years of age (Fig. 2). Children, and especially infants, were not normally treated in the medical ward and were not included in this material. However, the 15 patients under 15 years of age (2–14 years old) who were treated there all survived. This agrees with the national death registry statistics that did not reveal significantly elevated infant mortality during this period (28).

Among the 57 patients that died and were autopsied, pneumonia dominated the findings (49 of 55 evaluable records). The records were not completely comparable in classification and structure, and bacterial findings associated with the pneumonic picture were not specified. Severe underlying lung damage, presumably from viral pneumonitis, generally appeared with high proportions of hemorrhagic and bilateral disease; some cases were described as ‘pneumonia crouposa’ and interpreted as having a more inflammatory rather than purulent lung picture. The pathological findings described in the British pandemic influenza report appear similar those in the Uppsala material. The findings are described as bacterial, but not like common purulent lobular pneumonia, instead with massive edema, generalized infection and hemorrhage (42). These pathological findings together with the clinical description of significant cyanosis (42) support acute respiratory distress syndrome as the deathcause of the pandemic death cases.

Modern studies of autopsy material from Spanish flu cases and revision of autopsy series confirm the dominance of bacterial pneumonia as the cause of death (48). However, the severe viral damage of the airway epithelium from necrosis and edema supported the bacterial disease (49). Forty-five percent of the deaths noted in our material had additional involvement of nonpulmonary organs indicating generalized disease with multi-organ failure, which is in accordance with other materials from the time (42).

After the pandemic, the Swedish Red Cross assembled a committee to assess the effectiveness of the treatments used. In 1924, the committee concluded that none of the multitude of treatments attempted during the pandemic had any proven effects on the influenza (50). With today's knowledge, there is reason to believe that some of the regimes were indeed harmful. In addition to hemorrhagic disease, pulmonary edema was a frequent finding, and acetylsalicylic acid has been suggested as a contributing factor to both (40). Another questionable medical regime was the use of alcohol because high alcohol intake may decrease the immune response (51).

There are no Swedish studies assessing how the mortality rate was altered by nonpharmaceutical actions such as the closure of schools and dance halls, the banning of public funerals, isolation of infected, and so on. International studies, however, do show that implementation of nonpharmaceutical interventions resulted in reduced peak death rates of approximately 50% (52) and, if a combination of interventions was prolonged, the cumulative mortality could be significantly reduced (53). Unfortunately, interventions were rarely maintained for longer than 6 weeks after which the viral spread was renewed and the overall effect questioned (52).

A study of remote, small communities in the United States, such as colleges, institutions, and military facilities, concluded that strict compliance with isolation from early on and during the entire period of risk was a possible measure to avoid the spread of the virus (54), implying a possible way to further protect a population in a future pandemic setting. Sequestration of larger populations, however, has been difficult in the past and will continue to be so in the future.

The Spanish flu's economic effect on the Uppsala Academic Hospital, with doubled expenses during the period, was certainly a substantial burden for an already suffering hospital economy. This economic burden was not only caused by the Spanish flu. During the post-World War I years, business expenses were high. Combined with a shortage of fuel, groceries, and labor, this led to inflation and higher costs for the hospital. In 1920, the international business cycle changed and Sweden's gross domestic product (GDP) dropped by 35%; heavy spending for the hospital decreased and total expenses reversed toward pre-pandemic levels (55).

If a new pandemic with the same virulence as the Spanish flu were to affect the world and Uppsala today, the scenario would be different. Improved standards of living, vaccines, antibiotics, and antiviral drugs would decrease mortality, but crowding and rapid global movements would increase the transmission rate and speed the pandemic. It has been estimated that a new influenza pandemic with the same virulence as the Spanish flu would cause a global mortality rate of 51–81 million deaths (56), and that 4% of these would occur in Organisation for Economic Co-operation and Development (OECD) countries (56). Estimates conclude that the world's assembled costs would be close to $4.4 trillion (USD) or 12.6% of the global GDP (57). Although the impact would be greatest on non-OECD countries, Europe is expected to experience a 4.3% GDP reduction (58). In Uppsala County, with a present population of 342,000, this would mean an estimated mortality of 500–900 individuals and a final price tag of 840 million SEK ($120 million USD) – a perspective that would undisputedly paralyze society and the health care system.
